# A surgical resection of giant perianal mass secondary to complex anal fistula: a case report

**DOI:** 10.1093/jscr/rjae514

**Published:** 2024-08-23

**Authors:** Yu Fan, Zhun Yu, Cuizhu Xu, Jiali Wang, Ting Hu

**Affiliations:** Traditional Chinese Medicine Department, Third Affiliated Hospital of Changchun University of Chinese Medicine, Changchun 130000, China; Traditional Chinese Medicine Department, Third Affiliated Hospital of Changchun University of Chinese Medicine, Changchun 130000, China; Traditional Chinese Medicine Department, Third Affiliated Hospital of Changchun University of Chinese Medicine, Changchun 130000, China; Traditional Chinese Medicine Department, Third Affiliated Hospital of Changchun University of Chinese Medicine, Changchun 130000, China; Traditional Chinese Medicine Department, First Affiliated Hospital of Changchun University of Chinese Medicine, Changchun 130000, China

**Keywords:** complex anal fistula, giant perianal mass, anorectal operation, secondary mass, perianal malignant tumor

## Abstract

In complex anal fistula, the patient’s anus has multiple internal or external openings. The closure of the external opening can be broken again in other trips and another external opening. It is one of the refractory diseases in the field of anorectal surgery. In the treatment of a high recurrence rate, poor functional protection and other problems, surgical treatment such as incision and retention of sphincter hanging line, incision and suture internal opening drainage, fistula removal, and suture were used for different situations. After the failure of surgical treatment, it becomes chronic anal fistula or become cancerous tumor. In this case, the patient had a long time of illness, the mass was huge, the color was black, and it was suspected to be a secondary malignant tumor. This situation is extremely rare in clinical treatment. In our hospital, a case of complex anal fistula secondary to perianal huge tumor was found, and good therapeutic effect was achieved after complex anal fistula resection and tumor resection. In this case, the patient had complex anal fistula for about 5 years without timely treatment, and the perianal mass was rare and huge. It is shown by ultrasonography the secondary malignant tumor, which was extremely rare in clinical treatment. During the operation, it was found that three fistulas at the root of the tumor were connected with one of the anal fistulas. From the appearance, the tumor seems to have a malignant tendency. After surgical resection, the pathology was suppurative inflammation and granulation tissue hyperplasia. The patients recovered well after follow-up and did not relapse. This case report provides case reference and reference for clinical treatment of anorectal diseases.

## Case description

**Figure 1:**
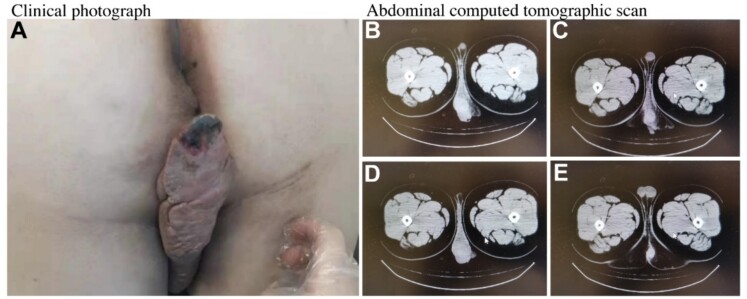
The soft mass and computed tomographic scan of the pelvis.

A 21-year-old man was admitted to our hospital with complex anal fistula and a painful anal mass. The anal lesion appeared 5 years earlier and grew slowly to the current size. Patient had perianal purulent secretions. The patient reported bleeding from the rectum after defecation and denied weight loss or fever. The patient noted no other symptoms. Physical examination revealed a 15 × 5 cm^2^ soft mass on the left side of the anus, as shown in Fig. 1A. The local skin edge is necrotic and the color is black. The patient underwent scrotal hydrosalpinx surgery 10 years ago. The anal position was normal, and an ulcer was seen at 2.5 cm from the anal margin in the direction of 1 o’ clock. The surface of the ulcer was hyperplastic, uneven, and no purulent secretion was found. The digital rectal examination was favorable, and a depression could be touched on the dentate line at 12 o’clock position. Other abnormal signs weren’t found. The recto copy was not performed because of the pain. The echo of ultrasonic examination is reduced. It may be secondary malignant tumor of anal fistulas. The computed tomographic scan of the pelvis showed a high density 8.8 × 5.6 cm^2^ mass extending to perineal position, as shown in Fig. 1B–E.

**Figure 2:**
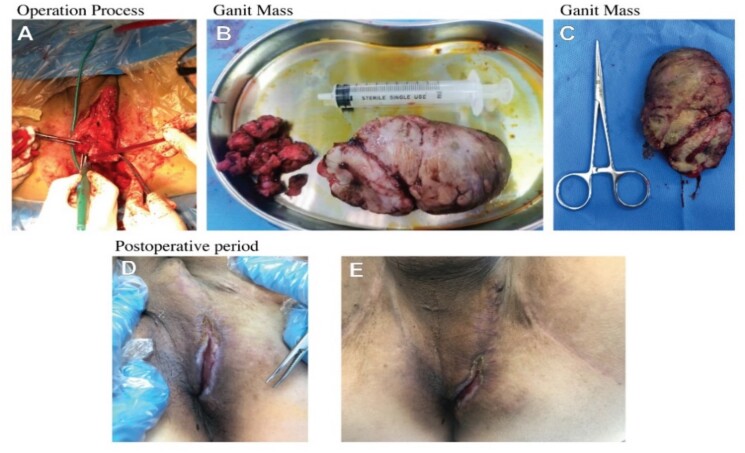
The operation process was to remove the tumor by electric knife.

**Figure 3:**
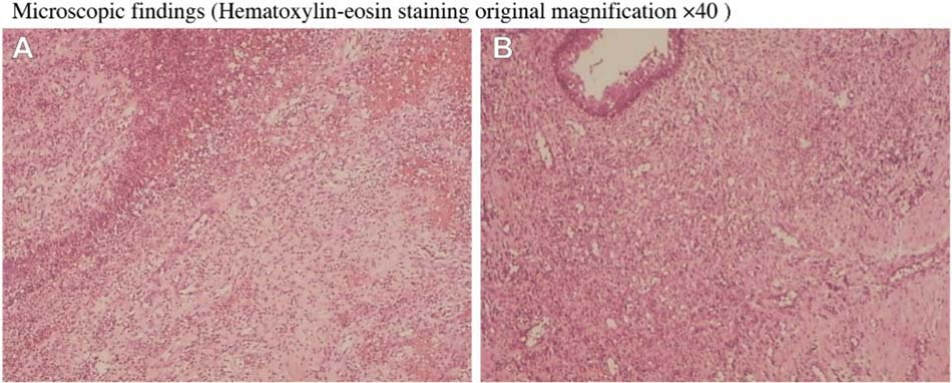
Microscopic findings.

The mass is very large and grows towards the perineum. The edge skin is damaged and the color is black, which does not exclude the malignant tumor secondary to anal fistula. During the operation, the diagnosis of complex anal fistula and perianal tumor was made. The operation process was to remove the tumor by electric knife, as shown in Fig. 2A–C. We cleaned the wound with normal saline, connected the three fistulas at the bottom of the tumor with the 1 o’ clock point ulcer, and cut the fistula and eliminate it. The other two fistulas passed through the deep part of the internal and external anal sphincter, and partial sphincter resection was performed to remove the fistula. After further exploration, no other residual cavity fistula was found. End-to-end mattress suture was performed on the deep end of the internal and external sphincter. Finally, the outer layer and skin flap suture were performed. There was no active bleeding and stenosis of the rectum and anal canal. The appearance of the anus looked flat, as shown in Fig. 2D and E. After surgical resection, pathological examination was performed. It was shown that suppurative inflammation, granulation tissue hyperplasia, and abscess formation in pathological report, as shown in Fig. 3A and B. (hematoxylin-eosin staining original magnification ×40).

## Discussion

Long-term chronic anal fistula is prone to cancer [1]. Some malignant tumors around the anus are often neglected, such as flat epithelial carcinoma, which often occurs in the anus or the skin around the anus covered by the anal epithelium. Some leiomyoma, gastrointestinal stromal tumors, are also easy to ignore, often confused with cancer. Most of the tumors grow slowly and do not form a clear intracavitary mass, which is often misdiagnosed as perianal or ischiorectal abscess and is repeatedly cut and drained. Finally, the correct diagnosis is made by pathological biopsy. Clinicians should be alert to perianal mucinous adenocarcinoma when there is more transparent jelly-like mucus in the fistula of patients with chronic anal fistula or when it is still delayed after formal treatment. In recent years, some scholars have reported that the development of perianal mucinous adenocarcinoma is slower than squamous cell carcinoma [2].

Some foreign scholars believe that chronic anal fistula rarely develops into cancer, and many scholars believe that there is basically no deterioration, just as duodenal ulcer is not cancerous. They believe that the so-called anal fistula carcinogenesis is mostly due to the invasion of anal canal cancer itself after the development of perianal tissue rupture [3, 4]. Some masses are caused by cancer metastasis. We still need to be alert to the possibility of deterioration after anal fistula and the growth of surrounding malignant tumors.

The treatment of complex anal fistula is not easy to improve. It takes a long time and even turns into chronic anal fistula, resulting in perianal masses. Patients often mistakenly think that it is hemorrhoids or other benign lesions. The patient’s tumor grew from the perianal fistula to the perineum, with a huge dimension and a black surface. It should be carefully identified with perianal malignant tumors. The excessive proliferation of the tumor is also very prone to malignant transformation. The early identification of many benign diseases and cancers is crucial for the good prognosis of many patients. In our case, the patient initially mistook the tumor for hemorrhoids and never visited the doctor, resulting in an increase in the size of the mass to a rare dimension. Fortunately, it is not cancer.

## Data Availability

All data that support the finding of this study are included in this manuscript and its supplementary information files.
